# Using the Stochastic Gradient Descent Optimization Algorithm on Estimating of Reactivity Ratios

**DOI:** 10.3390/ma14164764

**Published:** 2021-08-23

**Authors:** Iosif Sorin Fazakas-Anca, Arina Modrea, Sorin Vlase

**Affiliations:** 1 AGIMED Sovata, 545500 Sovata, Romania; d-mec@unitbv.ro; 2 Pharmacy, Science and Technology George Emil Palade Targu Mures, University of Medicine, 300134 Targu Mures, Romania; 3 Department of Mechanical Engineering, Transilvania University of Brasov, B-dul Eroilor 20, 500036 Brasov, Romania; 4 Romanian Academy of Technical Sciences, B-dul Dacia 26, 030167 Bucharest, Romania

**Keywords:** gradient descent, reactivity ratios, copolymerization

## Abstract

This paper describes an improved method of calculating reactivity ratios by applying the neuronal networks optimization algorithm, named gradient descent. The presented method is integral and has been compared to the following existing methods: Fineman–Ross, Tidwell–Mortimer, Kelen–Tüdös, extended Kelen–Tüdös and Error in Variable Methods. A comparison of the reactivity ratios that obtained different levels of conversions was made based on the Fisher criterion. The new calculation method for reactivity ratios shows better results than these other methods.

## 1. Introduction

Binary copolymerization is one of the essential techniques to obtain macromolecular compounds with controlled characteristics. To predict the copolymer composition and to control the properties, the calculation of reactivity ratios with higher accuracy becomes a critical goal. The most commonly used kinetic model in methods to calculate reactivity ratios is the terminal model, which is described by the following equations [1]:

$$\begin{array}{c} P_{n}-M_{1}^{*}+M_{1}\overset{k_{11}}{\to }P_{n+1}-M_{1}^{*}\\ P_{n}-M_{1}^{*}+M_{2}\overset{k_{12}}{\to }P_{n+1}-M_{2}^{*}\\ P_{n}-M_{2}^{*}+M_{2}\overset{k_{21}}{\to }P_{n+1}-M_{1}^{*}\\ P_{n}-M_{2}^{*}+M_{2}\overset{k_{22}}{\to }P_{n+1}-M_{2}^{*} \end{array}$$

where $P_{n}$—growing polymer chain, $M_{1}^{*}$, $M_{2}^{*}$—the active center on monomer 1 and on monomer 2, respectively, $k_{11}$, $k_{12}$, $k_{21}$, $k_{22}$—propagation rate constants.

The correlation of these four kinetic equations into an expression which can link the copolymer composition with the kinetic parameters can be carried out assuming that the monomer consumption in the initiation and the termination reaction can be neglected and rate of interchange of radicals is constant (1).

(1) $$k_{12}⎣M_{1}^{*}⎦\left[M_{2}\right]=k_{21}⎣M_{2}^{*}⎦\left[M_{1}\right]a=1,$$

Considering the two lemmas, the mathematical solution proposed by Mayo and Lewis [2] for the instantaneous consumption of monomers is described by Equation (2), and the integral form of this equation is described in Equation (3):

(2) $$\frac{d\left[M_{1}\right]}{d\left[M_{2}\right]}=\frac{\left[M_{1}\right]}{\left[M_{2}\right]}\frac{r_{1}\left[M_{1}\right]+M_{2}}{\left[M_{1}\right]+r_{2}\left[M_{2}\right]},$$

where:

(3) $$r_{1}=\frac{k_{12}}{k_{22}}\;\mathrm{and}\;r_{1}=\frac{k_{21}}{k_{22}}$$

and the integral form of this equation, described by Equation (4), has been proposed by Mayo and Lewis [2]:

(4) $$\log\frac{\left[M_{2}\right]}{\left[M_{20}\right]}=\frac{r_{2}}{1-r_{2}}\log\frac{\left[M_{20}\right]\left[M_{1}\right]}{\left[M_{10}\right]\left[M_{2}\right]}-\frac{1-r_{1}r_{2}}{\left(1-r_{1}\right)\left(1-r_{2}\right)}\log\frac{\left(r_{1}-1\right)\frac{\left[M_{1}\right]}{\left[M_{2}\right]}-r_{2}+1}{\left(r_{1}-1\right)\frac{\left[M_{10}\right]}{\left[M_{20}\right]}-r_{2}+1}$$

Determining the reactivity ratios with relation (4) is difficult, but is easier to use if it has been transformed in the following relation:

(5) $$r_{2}=\frac{\log\frac{M_{2}^{o}}{M_{2}}-\frac{1}{p}\log\frac{1-p\frac{M_{1}}{M_{2}}}{1-p\frac{M_{1}^{o}}{M_{2}^{o}}}}{\log\frac{M_{1}^{o}}{M_{1}}+\frac{1}{p}\log\frac{1-p\frac{M_{1}}{M_{2}}}{1-p\frac{M_{1}^{o}}{M_{2}^{o}}}},$$

where:

(6) $$p=\frac{1-r_{1}}{1-r_{2}},$$

$⎣M_{1}^{o}⎦$, $⎣M_{2}^{o}⎦$—the initial molar fraction of monomers in feed, $\left[M_{1}\right]$, $\left[M_{2}\right]$—molar fraction of monomers in feed at given conversion.

Considering the same lemmas, Wall [3] and Skeist [4] propose the use of the following mathematical equation to correlate the kinetic parameters with the instantaneous copolymer composition:

(7) $$\frac{\Delta M_{1}}{\Delta M_{1}+\Delta M_{2}}=m_{1}=\frac{r_{1}f_{1}^{2}+f_{1}f_{2}}{r_{1}f_{1}^{2}+2f_{1}f_{2}+r_{2}f_{2}^{2}},$$

where $f_{1}$ and $f_{2}$ are the molar fractions of monomer 1 and 2, respectively, in feed; $\Delta M_{1}$ and $\Delta M_{2}$ are the amounts of the corresponding components which enter the polymer in a differential time interval; *m*_1_ is the mole fraction of the first component in the polymer formed during the differential time interval and $r_{1}$, $r_{2}$ are reactivity ratios for monomers 1 and 2, respectively. Equation (7), proposed by Wall and Skeist, is a nested equation of the Mayo–Lewis Equation (2).

The integration form of Equation (7) was made by Meyer and Lowry [5] and is given by the following equation:

(8) $$\frac{\left[M_{1}\right]+\left[M_{2}\right]}{\left[M_{1}^{0}\right]+\left[M_{1}^{0}\right]}={\left(\frac{f_{1}}{f_{1}^{0}}\right)}^{\alpha }{\left(\frac{f_{2}}{f_{2}^{0}}\right)}^{\beta }{\left(\frac{f_{1}^{0}-\delta }{f_{1}-\delta }\right)}^{\gamma },$$

where:

(9) $$f_{1}=\frac{\left[M_{1}\right]}{\left[M_{1}\right]+\left[M_{2}\right]}=1-f_{2},$$

(10) $$\alpha =\frac{r_{2}}{1-r_{2}}\;;\;\beta =\frac{r_{1}}{1-r_{1}}\;;\;\gamma =\frac{1-r_{1}r_{2}}{\left(1-r_{1}\right)\left(1-r_{2}\right)}\;;\;\delta =\frac{1-r_{2}}{2-r_{1}-r_{2}},$$

Over time, based on these mathematical models, several methods have been created to calculate reactivity ratios. The proposed methods for calculating reactivity ratios are classified as:

- In differential methods using Equations (2) and (7) as models, where the conversions are usually <10% and the calculated composition of the copolymer is the instantaneous one;
- In integral methods using Equations (4), (5) and (8) as models, where the conversions are usually >10% and the calculated composition of the copolymer is the global composition.

Another classification type of the methods for calculating the reactivity ratios can be made according to the mathematical way by which the values of the variables *r*_1_ and *r*_2_ are obtained, as follows: linear methods and nonlinear methods.

One of the commonly used differential methods is the graphical method proposed by Fineman–Ross (FR for Equation (10) and r-FR for Equation (11)) [6] which is linearized in Equation (2) and is described by the following equations:

(11) $$\frac{F}{f}\left(f-1\right)=r_{1}\frac{F^{2}}{f}-r_{2},$$

(12) $$\frac{f-1}{f}=-r_{2}\frac{f}{F^{2}}+r_{1},$$

where

(13) $$f=\frac{m_{1}}{m_{2}}\approx \frac{dM_{1}}{dM_{2}}\;;\;F=\frac{m_{1}}{m_{2}},$$

When using the Fineman–Ross method, the slope of Equation (11) and the intercept of Equation (12) give the value for *r*_1_ and, respectively, the intercept of Equation (11) and the slope of Equation (12) give the value for *r*_2_. The distribution of the calculated points using Equations (11) and (12) is not uniform along the line. These calculated points are crowded in the area of origin of the coordinate system, and due to experimental errors, can easily lead to errors in estimating the values of the slope and the intercept and obviously the values of the reactivity ratios are affected.

One solution to eliminate this disadvantage of the Fineman–Ross method was proposed by Kelen–Tüdös (KT) [7,8]. This method is also based on linearization of Equation (2) and is described by the following mathematical equations:

(14) $$\frac{G}{a+F}=\left(r_{1}+\frac{r_{2}}{\alpha }\right)\frac{F}{\alpha +F}-\frac{r_{2}}{\alpha },$$

where:

(15) $$\alpha =\sqrt{F_{\min}\cdot F_{\max}},$$

(16) $$G=x\frac{y-1}{y}\;\mathrm{and}\;F=x\frac{x^{2}}{y}$$

(17) $$x=\frac{M_{1}}{M_{2}}\;\mathrm{and}\;y=\frac{dM_{1}}{dM_{2}}\approx \frac{m_{1}}{m_{2}}$$

The KT method only gives one solution of reactivity ratios and the calculated points are homogenously distributed along the line.

Calculating the reactivity ratios using the linearization technique of the KT method was extended by the same authors [9,10] for experimental data obtained at high conversions. For this reason, the mathematical Equation (8) is rewritten as follows:

(18) $$\frac{z\left(y-1\right)}{\alpha z^{2}+y}=\left(r_{1}+\frac{r_{2}}{\alpha }\right)\frac{y}{az^{2}+y}-\frac{r_{2}}{\alpha },$$

where:

(19) $$z=\frac{\log\frac{M_{1}}{M_{10}}}{\log\frac{M_{2}}{M_{20}}}=\frac{\log\left[1-\frac{y}{x_{0}}\log\left(1-P_{n}\frac{\bar{\alpha }+x_{0}}{\bar{\bar{\alpha }}+y}\right)\right]}{\log\left(1-P_{n}\frac{\bar{\alpha }+x_{0}}{\bar{\bar{\alpha }}+y}\right)},$$

(20) $$\bar{\alpha }=\frac{\mu_{1}}{\mu_{2}},$$

(21) $$x_{0}=\frac{M_{10}}{M_{20}}\;\mathrm{and}\;y=\frac{m_{1}}{m_{2}}$$

where the 0 index refers to the initial concentration of monomer i, *α* has the same mathematical form as Equation (8), Pn weight percent conversion, *μ*—molecular weight of monomers. Under this mathematical form, the method is known as the extended Kelen–Tüdös method (eKT).

For both methods of calculating the reactivity ratios proposed by Kelen and Tudos (KT, e-KT), the value of *r*_2_ is obtained from the value of the intercept multiplied by the correction factor α and the value of *r*_1_ is obtained from the value of the slope which decreases the value of the intercept.

The transformation of the Mayo–Lewis Equation (2) into a weighted (Equations (14) and (18)) or unweighted linear form (Equations (11) and (12)) admits the existence of a dependent variable y, the left hand of equations and an independent variable x, the term that multiplies the reactivity ratio to the right of the equations. The reactivity ratios calculated with linear methods represent the slope and intercept of the line, and to obtain these two parameters, linear regression using ordinary least squares methods (OLS) is carried out. To estimate the best values of reactivity ratios using OLS, the variables x and y must meet the Gauss–Markov assumptions, which are:

- The dependence between y and x variables must be linear and to have random errors. Non-linearity gives a wrong estimation of reactivity ratios;
- For independent variable x, the expected error term is zero, otherwise the intercept is biased;
- The covariance of errors for all independent variables x is constant and represents the measurement of the model uncertainty. If the covariance of errors is not constant, the estimated reactivity ratios are less precise, which increases the likelihood of being further from the correct values;
- The standard hypothesis in linear regression is that the independent variable x is not dependent on the dependent variable y or, put another way, each independent variable x is uncorrelated with the error terms. In the linear methods of reactivity ratio calculation, the variables x and y are correlated; for this reason, it is possible to obtain biased or inconsistent reactivity ratios;
- All independent variables x must by collinear, otherwise the calculated reactivity ratios with OLS will have big errors.

Considering those assumptions presented above, the reactivity ratios obtained using linear methods must be viewed with caution in terms of their quality if supplementary information about measurement errors does not exist.

The Tidwell–Mortimer (TM) [9] method is a differential optimization method based on the modified Gauss–Newton nonlinear least-square algorithm. They developed a method of calculating reactivity ratios from Equation (4) and derived the following relationship:

(22) $$m_{21}=G_{i}^{j}+\left(r_{1}^{0}-r_{1}^{j}\right)\frac{\partial G_{i}^{j}}{\partial r_{1}}+\left(r_{2}^{0}-r_{2}^{j}\right)\frac{\partial G_{i}^{j}}{\partial r_{2}}+\varepsilon_{i},$$

where:

(23) $$G^{j}=\frac{r_{2}^{j}f_{2}^{2}+f_{1}f_{2}}{r_{2}^{j}f_{2}^{2}+2f_{1}f_{2}+r_{1}^{j}f_{2}^{2}},$$

*i* is the number of the experimental run, *j* is number of the estimation set and $r_{1}^{0}$, $r_{2}^{0}$ are the expectation values of $r_{1}^{j}$ and $r_{2}^{j}$ respectively.

Thus, if the difference between the molar fraction measured experimentally and the one calculated using the Wall equation using Equation (6):

(24) $$d_{i}=m_{2i}-G_{i}^{j}=\beta_{1}\frac{\partial G_{i}^{j}}{\partial r_{1}}+\beta_{2}\frac{\partial G_{i}^{j}}{\partial r_{2}}+\varepsilon_{i},$$

then estimates, ${\hat{\beta }}_{1}$, ${\hat{\beta }}_{2}$ of the smallest squares of $\beta_{1}$ and $\beta_{2}$ provide the necessary corrections so that the new values of $r_{1}^{j}$ and $r_{2}^{j}$ given by

(25) $$r_{1}^{j+1}=r_{1}^{j}+{\hat{\beta }}_{1},$$

(26) $$r_{2}^{j+1}=r_{2}^{j}+{\hat{\beta }}_{2},$$

introduced in Equation (10) should lead to a decrease in value the $\sum^{\;}{\left(d_{i}\right)}^{2}$. This method is known as the Gauss–Newton optimization method.

Although integral methods for calculating reactivation ratios have existed for a long time, their use is limited and reduced to a few simpler methods based either on the linearization of integral equations or on the minimization of errors in one variable or both variables.

The Error in Variable Method (EVM) is a concept for calculating reactivity ratios rather than a method itself because there are many variants of it [11,12,13,14,15,16,17,18,19]. When this concept was first used to determine the reactivity ratios, it was admitted that the measurement errors are only in one of the variables [11]; later, the concept used the analysis of errors in both variables [12,13,14,15,16,17,18,19]. The EVM variant proposed by van der Meer et al. [12], Patino-Leal et al. [13], and Hautus et al. [14] uses the mathematical model given by Equation (3); Yamada et al. [10] use Equation 5 as a mathematical model and Kazemi [17] use the Meyer–Lowry model (Equation (6)).

One of the EVM variant methods is the one proposed by Chee and Ng [20], which uses the integral form proposed by Mayo–Lewis (3) as a mathematical model and is based on the minimization of the residual weighted sum for *r*_2_, defined by the following relation:

(27) $$S={\sum W\left(r_{2}-r_{2}^{e}\right)}^{2},$$

where:

(28) $$W=\frac{1}{Var(r_{2}-r_{2}^{pe})}=\frac{1}{Var(f)},$$

(29) $$\begin{array}{l} Var(f)={\left(\frac{\partial f}{\partial x}\right)}^{2}Var(x)+{\left(\frac{\partial f}{\partial y}\right)}^{2}Var(y)+{\left(\frac{\partial f}{\partial P_{n}}\right)}^{2}Var(P_{n})+2\left(\frac{\partial f}{\partial x}\right)\left(\frac{\partial f}{\partial y}\right)Cov(x,y)\\ +2\left(\frac{\partial f}{\partial y}\right)\left(\frac{\partial f}{\partial P_{n}}\right)Cov(y,P_{n})+2\left(\frac{\partial f}{\partial x}\right)\left(\frac{\partial f}{\partial P_{n}}\right)Cov(x,P_{n}) \end{array}$$

(30) $$x=\frac{M_{10}}{1-M_{10}}\;\mathrm{and}\;y=\frac{m_{1}}{1-m_{1}}$$

(31) $$Var(x)={\left(1+x\right)}^{4}\sigma_{M}^{2}$$

(32) $$Var(y)={\left(1+y\right)}^{4}\sigma_{M}^{2}$$

(33) $$Var(P_{n})=P_{n}\left\{{\left(\frac{\sigma_{P}}{P_{W}}\right)}^{2}+{\left(1-\bar{\alpha }\right)}^{2}\left[{\left(\frac{x}{1+\bar{\alpha }x}\right)}^{2}{\left(\frac{\sigma_{M}}{M_{10}}\right)}^{2}+{\left(\frac{y}{1+\bar{\alpha }y}\right)}^{2}{\left(\frac{\sigma_{m}}{M_{1}}\right)}^{2}\right]\right\}$$

(34) Cov(x,y)=0

(35) $$Cov(y,P_{n})=\left(\frac{\partial P_{n}}{\partial y}\right)Var(y)$$

(36) $$Cov(x,P_{n})=\left(\frac{\partial P_{n}}{\partial x}\right)Var(x)$$

$r_{2}^{e}$—the value of $r_{2}$ estimated with Equation (3), Pn weight percent conversion, *σ*—standard deviation of $M_{10}$, $m_{1}$ and $P_{n}$, *μ*—molecular weight of monomers.

The improved method proposed below is based on numerical integration step by step of a differential equation and uses the gradient descent optimization algorithm.

## 2. Material and Methods

A simplified algorithm used in the processes of optimizing neural networks [21] and machine learning [22] is that of the stochastic gradient descent (GD). This optimization algorithm was adapted for the process of the calculation of the reactivity ratio for a terminal model of copolymerization. In this procedure, it is assumed that the Wall –Skeist Equation (4) is valid and the experimental error is independent and has a common variance. The calculation principle of this algorithm consists of determining the parameters *r*_1_, *r*_2_ of a function *m*_2*i*_ (Equation (9)) by minimizing the cost of the function.

The calculation procedure of the descending gradient algorithm consists of the following steps:

- Initialize the value of reactivity ratios, $r_{1}^{j}$, $r_{2}^{j}$ with values obtained with the Fineman–Ross method;
- By introducing $r_{1}^{j}$, $r_{2}^{j}$ values into Equation (8), calculate $m_{2i}$ by numerical integration for each experimental point until the specific conversion has been reached using the numerical integration algorithm proposed by Kazemi [17,18];
- The cost of the coefficients $r_{1}^{j}$, $r_{2}^{j}$ is evaluated with the following equation, Equation (15):
  (37) $$\cos t^{j}=\left(m_{2i}^{j\;\exp}-m_{2i}^{j\;calc}\right)$$
  where $m_{2i}^{j\exp}$ is the experimentally determined copolymer composition, $m_{2i}^{jcalc}$ is the copolymer composition calculated with Equation (2), and *j* is the number of the calculation step.

- The partial derivative of the cost function (δ) according to *r*_1_ and *r*_2_ is calculated to determine the direction of evolution of the parameters $r_{1}^{j}$, $r_{2}^{j}$. Equations (35) and (36):
  (38) $$\delta_{1}^{j}=\frac{1}{n}\sum \left(G_{i}^{j}-m_{2i}^{j}\right)\frac{\partial G_{i}^{j}}{\partial r_{1}^{j}}$$
  (39) $$\delta_{2}^{j}=\frac{1}{n}\sum \left(G_{i}^{j}-m_{2i}^{j}\right)\frac{\partial G_{i}^{j}}{\partial r_{2}^{j}}$$
  where *n* is the number of experimental sets.
- The new values of the reactivity ratios $r_{1}^{j+1}$, $r_{2}^{j+1}$ are calculated with Equations (40) and (41):
  (40) $$r_{1}^{j+1}=r_{1}^{j}-\left(\alpha \delta_{1}\right)$$
  (41) $$r_{2}^{j+1}=r_{2}^{j}-\left(\alpha \delta_{2}\right)$$
  where *α* is the search step which has a small positive value *α* ∈[0,1], in this case, *α* = 0.1.
- The error of the method is determined using Equation (42):
  (42) $$err=\left|\frac{1-\cos t^{j}}{\cos t^{j-1}}\right|$$
- If the error is higher than a preset value (err = 1 × 10^−15^ in this case), then the calculation is resumed with new coefficients $r_{1}^{j+1}$, $r_{2}^{j+1}$, or else the calculation will stop.

The evaluation of the quality of the reactivity ratios obtained is performed using the Fisher criterion (F), whose formula shown below.

(43) $$F_{c}=\sqrt{\frac{\sum_{j=1}^{n}\sum_{i=1}^{p}\left(m_{i}^{j(e)}-m_{i}^{j(c)}\right)}{n(p-n+1)}}$$

where *F_c_* is the value of the Fisher criterion, *n* is the number of monomers used in copolymerization and *p* is the number of the experimental data set. Thus, $m_{i}^{j(e)}$ is the molar fraction of monomer “*i*” from copolymer for “*j*” experimental data set, and $m_{i}^{j(c)}$ is the molar fraction of monomer “*i*” calculated based on a mathematical model for the experiment “*j*”.

As can be seen from Equation (43), the lower the value of the Fisher criterion, the closer the values of reactivity ratios are to the true value.

The analysis of the quality of the methods for calculating the reactivity ratios considered in this paper was carried out by imposing the conditions presented in Table 1. The choice of reactivity ratios used in the qualitative analysis was made based on the following criteria: (*r*_1_ × *r*_2_): *r*_1_ × *r*_2_ ≈ 0; *r*_1_ × *r*_2_ ∊ [0,1] one < 0.5 other >0.5; *r*_1_ × *r*_2_ > 1.

For these imposed conditions, the initial compositions of the monomer mixture between 0–1 and conversions were normalized and randomly generated in the imposed intervals given in Table 1. With these data, for the given conversions the composition of the copolymer was calculated using numerical integration of Mayo–Lewis equation until the specific conversion for each point was reached. In Table 2, Table 3 and Table 4, the obtained data is shown, where LC is low conversion, MC is medium conversion and HC is high conversion and numbers refer to 1 for *r*_1_ = 0.05, *r*_2_ = 0.5., 2 for pairs *r*_1_ = 0.8, *r*_2_ = 1.8 and 3 for *r*_1_ = 0.4, *t*_2_ = 0.8.

Further, the analysis of the quality of the reactivity ratios obtained with the described methods was also carried out for the published experimental data with respect to the following conditions: at low conversion (1.0–9.5%) for the copolymerization of n-butyl methacrylate with n-butyl acrylate [23] in bulk at 80 °C initiated by BPO, at medium conversion (14.0–16.5%) for the copolymerization of 2-isopropenyl-2-oxazoline with methyl methacrylate [24] in acetonitrile at 70 °C initiated by AIBN, and at high conversion (10.9–55.0%) for the copolymerization of N-(4-carboxyphenyl) maleimide (NCPM) with N-vinyl-2-pyrrolidone [25] (NVP) in dimethylformamide at 90 °C.

The GD method was written in the Python 3.7 programming language.

## 3. Results

After processing the initial data presented in Table 2, Table 3 and Table 4, the following reactivity ratios were obtained, which are presented in Table 5, Table 6 and Table 7 for low conversion, in Table 8, Table 9 and Table 10 for medium conversion and in Table 11, Table 12 and Table 13 for high conversion, and also, in Table 14, Table 15 and Table 16 the reactivity ratios obtained with the analyzed methods and the reported reactivity ratios for the published experimental data are presented.

## 4. Discussion

**Figure 1:**
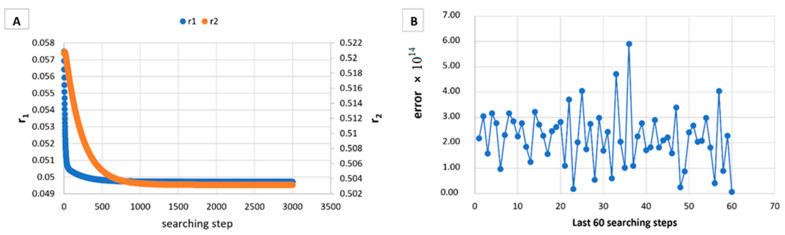
Evolution of reactivity ratio searching (**A**) and error for last 60 searching steps (**B**) with GD method for *r*_1_ = 0.05, *r*_2_ = 0.5 and *P_n_* ∊ (1–10%).

**Figure 2:**
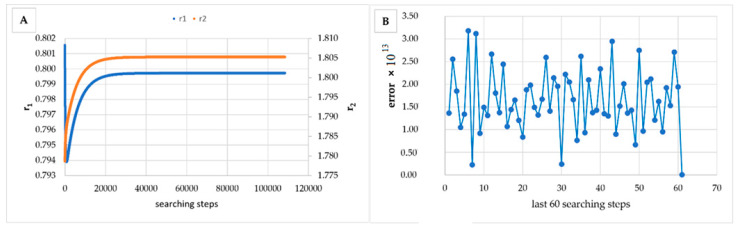
Evolution of reactivity ratio searching (**A**) and error for last 60 searching steps (**B**) with GD method for *r*_1_ = 0.8, *r*_2_ = 1.8 and *P_n_* ∊ (1–10%).

**Figure 3:**
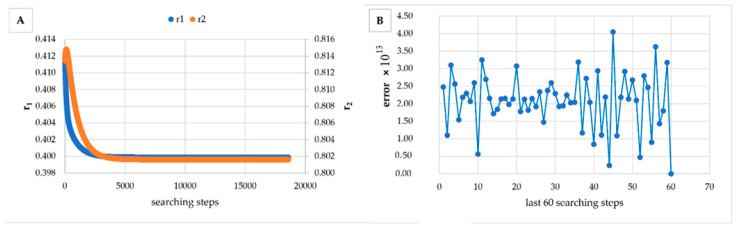
Evolution of reactivity ratio searching (**A**) and error for last 60 searching steps (**B**) with GD method for *r*_1_ = 0.4, *r*_2_ = 0.8 and *P_n_* ∊ (1–10%).

**Figure 4:**
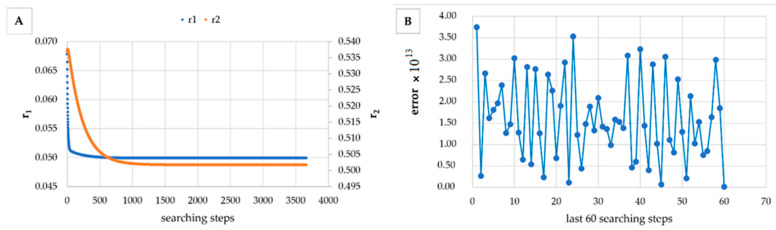
Evolution of reactivity ratio searching (**A**) and error for last 60 searching steps (**B**) with GD method for *r*_1_ = 0.05, *r*_2_ = 0.5 and *P_n_* ∊ (10–25%).

**Figure 5:**
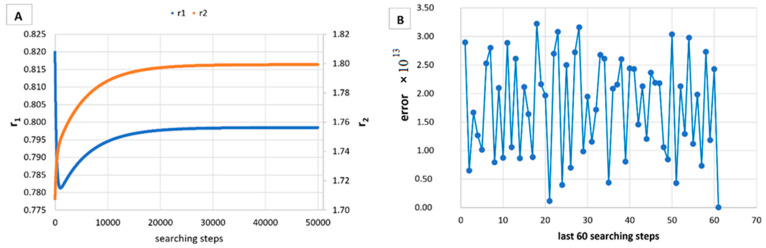
Evolution of reactivity ratio searching (**A**) and error for last 60 searching steps (**B**) with GD method for *r*_1_ = 0.8, *r*_2_ = 1.8 and *P_n_* ∊ (10–25%).

**Figure 6:**
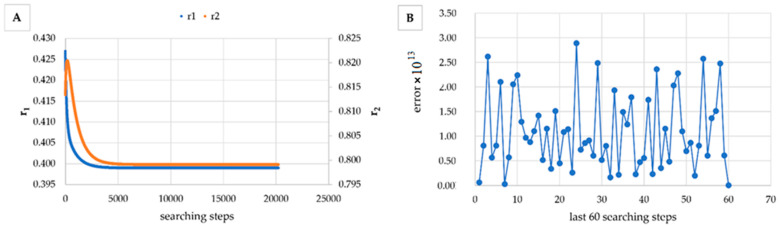
Evolution of reactivity ratio searching (**A**) and error for last 60 searching steps (**B**) with GD method for *r*_1_ = 0.4, *r*_2_ = 0.8 and *P_n_* ∊ (10–25%).

**Figure 7:**
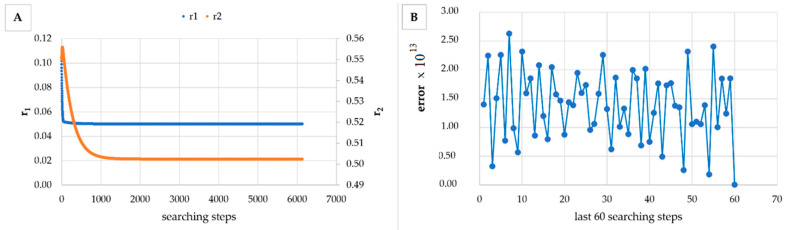
Evolution of reactivity ratio searching (**A**) and error for last 60 searching steps (**B**) with GD method for *r*_1_ = 0.05, *r*_2_ = 0.5 and *P_n_* ∊ (30–55%).

**Figure 8:**
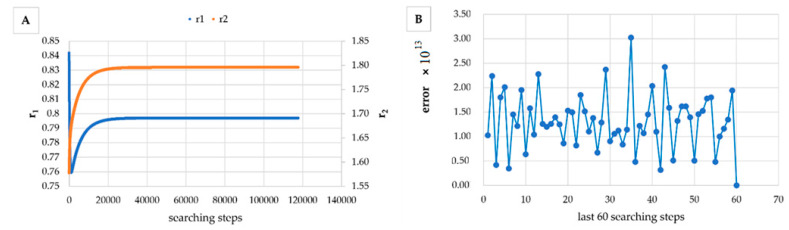
Evolution of reactivity ratio searching (**A**) and error for last 60 searching steps (**B**) with GD method for *r*_1_ = 0.8, *r*_2_ = 1.8 and *P_n_* ∊ (30–55%).

**Figure 9:**
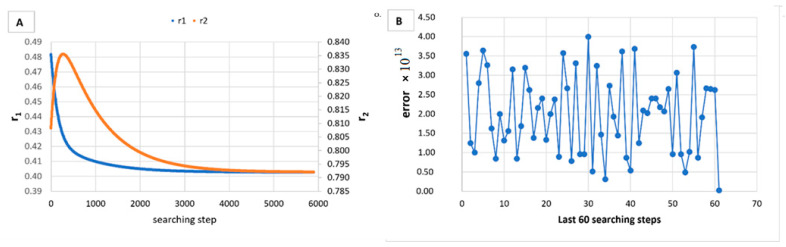
Evolution of reactivity ratio searching (**A**) and error for last 60 searching steps (**B**) with GD method for *r*_1_ = 0.4, *r*_2_ = 0.8 and *P_n_* ∊ (30–55%).

However, in Table 5, Table 6, Table 7, Table 8, Table 9, Table 10, Table 11, Table 12 and Table 13 it can be easily seen that in all cases, the integral method GD has the lowest bias compared to the initial conditions imposed, as well as the lowest values of the Fisher criterion. The e-KT method gives good results, with one exception at high conversion for values of *r*_1_ = 0.05, *r*_2_ = 0.5. The reason why by the e-KT method for the reactivity ratios *r*_1_ = 0.05, *r*_2_ = 0.5 at high conversions gives wrong values is due to the limitations of the method. This method can be used successful up to 40% conversions as well as to the errors of the logarithm function of around of 0 values. More details have been described by Tudos et al. [10]. The EVM method proposed by Chee and Ng [19] is usable, with good results in a few cases. The limitation of the EVM variant proposed by Chee and Ng [19] is due to the mathematical model used, because when one of the reactivity ratios is very close to zero and the conversion is high, the factor of logarithm can take negative values; for this reason, the results are erroneous. In Figure 1, Figure 2, Figure 3, Figure 4, Figure 5, Figure 6, Figure 7, Figure 8 and Figure 9, the evolution of reactivity ratios values is depicted, with searching steps and error value (Equation (39)) evolution for last 60 searching steps for the GD method for data given in Table 5, Table 6, Table 7, Table 8, Table 9, Table 10, Table 11, Table 12 and Table 13.

As can be seen from Figure 1, Figure 2, Figure 3, Figure 4, Figure 5, Figure 6, Figure 7, Figure 8 and Figure 9, the best values of reactivity ratios obtained with the GD method are strongly dependent on the initial value of the reactivity ratios used in the optimization process. Additionally, the values of the reactivity ratios obtained by descent gradient optimization are global minimums. Because the convergence of the err function (Equation (39)) is small, there are situations in which the GD method requires a high number of search steps to reach the proposed threshold value (10^−15^), which leads to a longer search time. Reducing the threshold value imposed will lead to a decrease in the calculation time but can also reduce the accuracy of the result.

It is also well known that from a statistical point of view, all combinations of pairs of reactivity ratios that can be obtained from within the 95% confidence domain satisfy the experimental data for the chosen mathematical model. For this reason, the reliable domains for the calculation methods taken in the analysis for the three initial conditions are drawn in Figure 10, Figure 11, Figure 12, Figure 13, Figure 14, Figure 15, Figure 16, Figure 17, Figure 18, Figure 19, Figure 20, Figure 21 and Figure 22, and published data are drawn in Figure 23, Figure 24 and Figure 25.

The JCR domains that do not appear in these figures are so large that they make the other JCRs no longer observable.

Although in all cases regardless of the initial conversions, the bias between the calculated value of the reactivity ratios by the analyzed methods and the target value are small, the JCR domains show their quality difference.

In the case of conversions below 10% and *r*_1_ = 0.05, *r*_2_ = 0.5 the r-FR method, through its reliable JCR, can also admit the possibility of aberrant solutions, i.e., negative reactivity ratios that are not allowed kinetically by their definition. The GD and e-KT methods have the lowest reliable and practical identical JCR in the case of experimental data with conversions of less than 10%.

**Figure 13:**
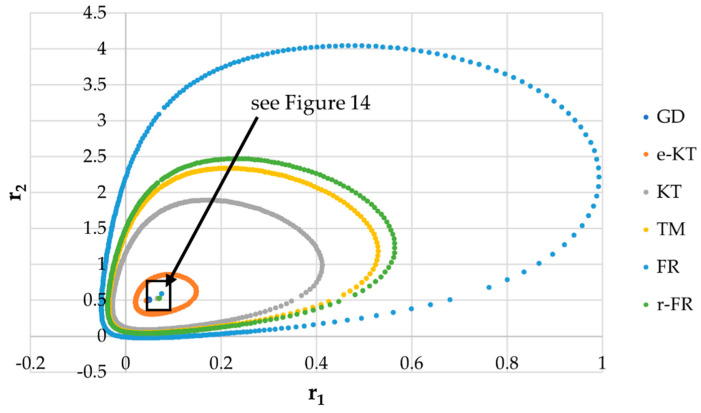
The JCR for initial conditions *r*_1_ = 0.05, *r*_2_ = 0.5, *P_n_* ∊ (10–25%).

**Figure 14:**
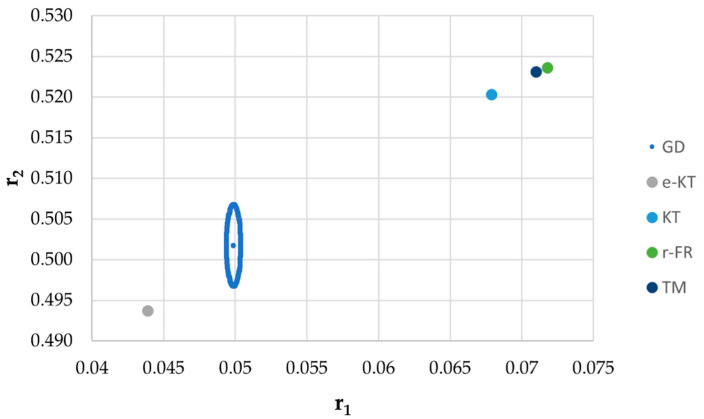
Details of JCR for GD given in Figure 13.

**Figure 15:**
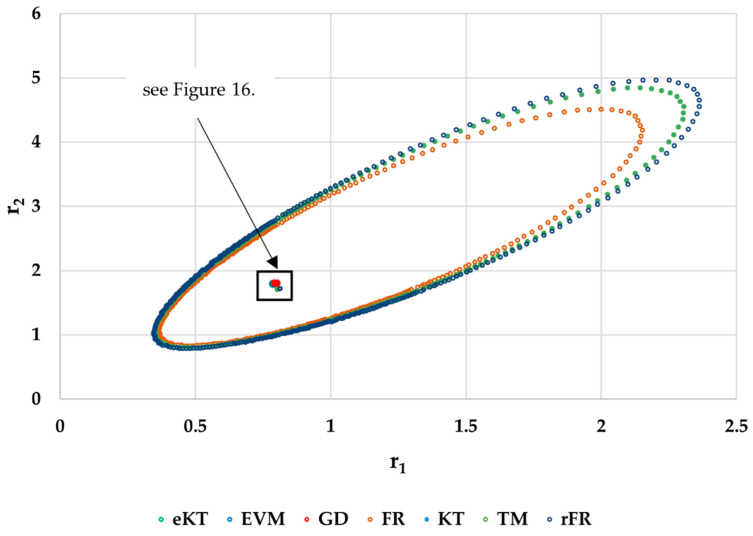
The JCR for initial conditions *r*_1_ = 0.80, *r*_2_ = 1.80, *P_n_* ∊ (10–25%).

**Figure 16:**
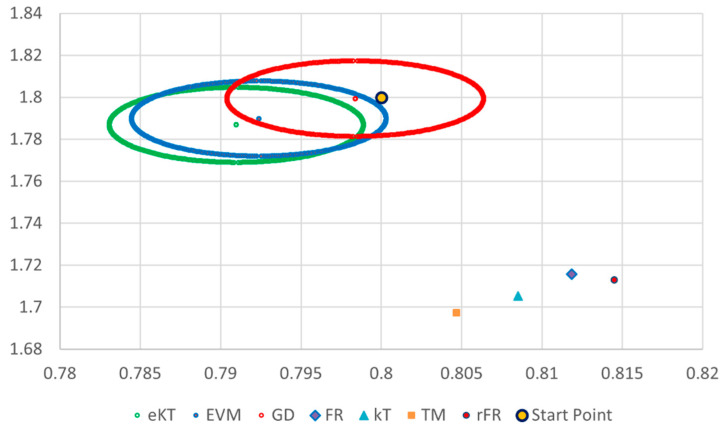
Details of JCR for GD, EVM and e-KT given in Figure 15.

**Figure 17:**
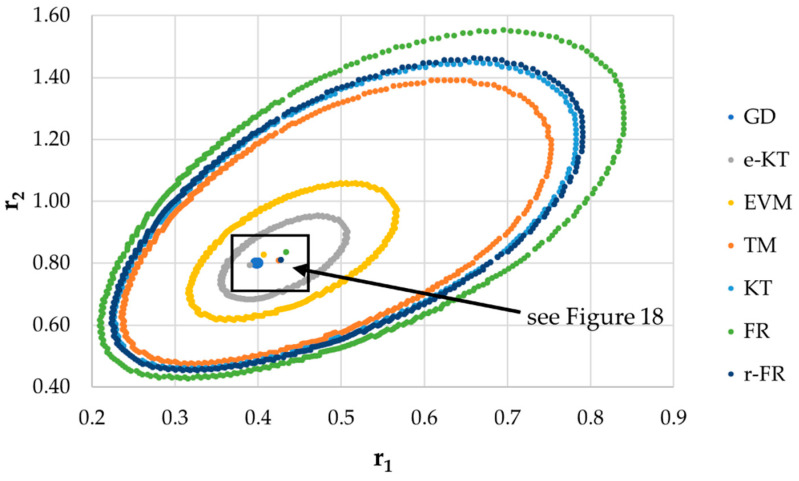
The JCR for initial conditions *r*_1_ = 0.4, *r*_2_ = 0.8, *P_n_* ∊ (10–25%).

**Figure 18:**
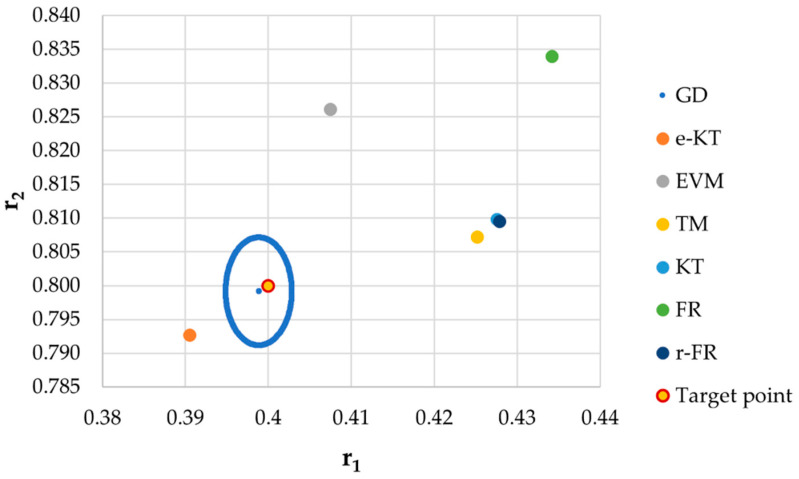
Details of JCR for GD given in Figure 17.

In the case of the conversion range between 10–25%, a good separation of the quality of the results between the differential and the integral methods can be made. This is observable both by the value of criterion *F_c_* and by the size of the JCR domain shown in Figure 13, Figure 14, Figure 15, Figure 16, Figure 17 and Figure 18. It should be noted that among the integral calculation methods for *r*_1_ = 0.8 and *r*_2_ = 1.8, although they have a close value of criterion F^c^ and the confidence domains are small, GD is the only method that admits the target value within its JCR domain. Additionally, it should be noted that the reactivity ratios obtained by the methods presented are outside the JCR of the GD method. It is obvious that at high conversions over 30%, differential methods give erroneous values and large areas of JCR. In this case, there is a good separation of the quality of the results obtained between the GD and e-KT method. Surprisingly, the EVM method analyzed here, although an integral method, does not give acceptable results.

Comparing the values of criterion F^c^ and the size of the 95% confidence regions, it can be said that there is a good correlation between them; therefore, criterion F^c^ can be used as an indicator of the quality of reactivity ratios.

## 5. Conclusions

It was observed that there is a good correlation between the value of criterion F^c^ and the size of the JCR domain. For this reason, criterion F^c^ could be used as an indicator of the quality of reactivity ratios. Regardless of the value of the conversions, the integral methods gave better results than the differential methods. From these analyzed integral methods, the GD method presented here can be successfully used to obtain reactivity ratios for experimental data that obtained up to 50–55% conversions.

## Figures and Tables

**Figure 10:**
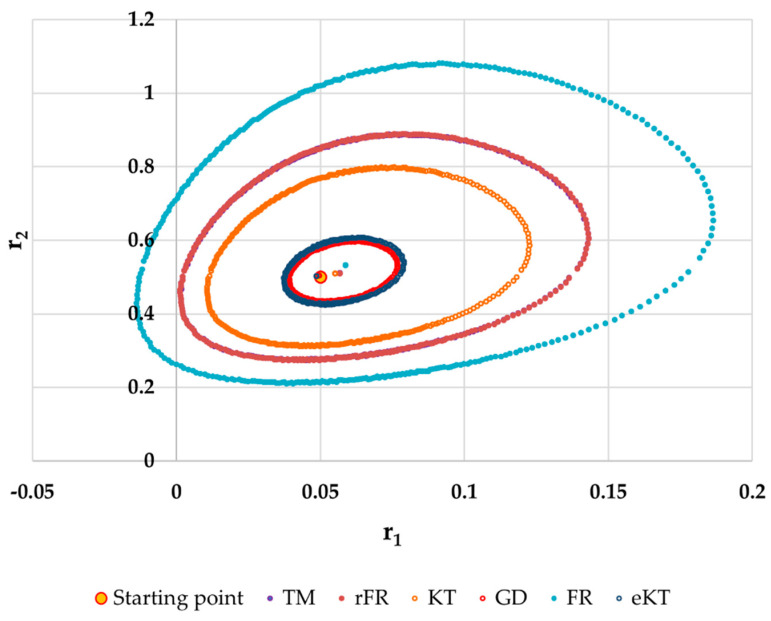
The JCR for following initial conditions *r*_1_ = 0.05, *r*_2_ = 0.50 *P_n_* ∊ (1–10%).

**Figure 11:**
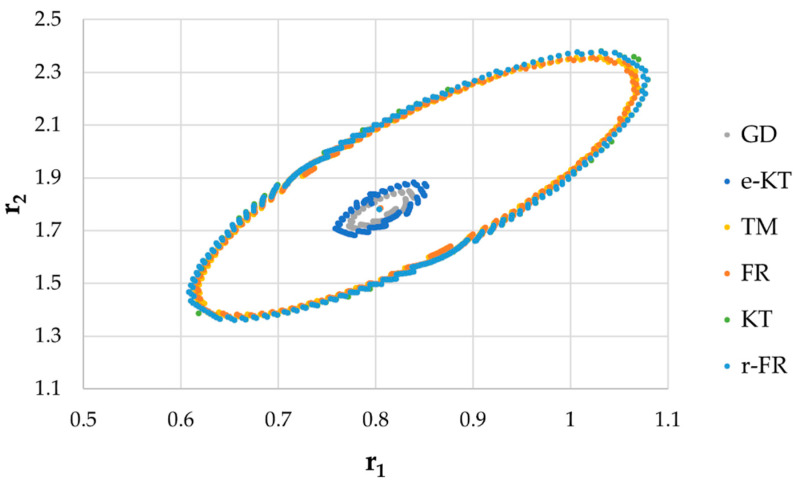
The JCR for following initial conditions *r*_1_ = 0.8, *r*_2_ = 1.8 *P_n_* ∊ (1–10%).

**Figure 12:**
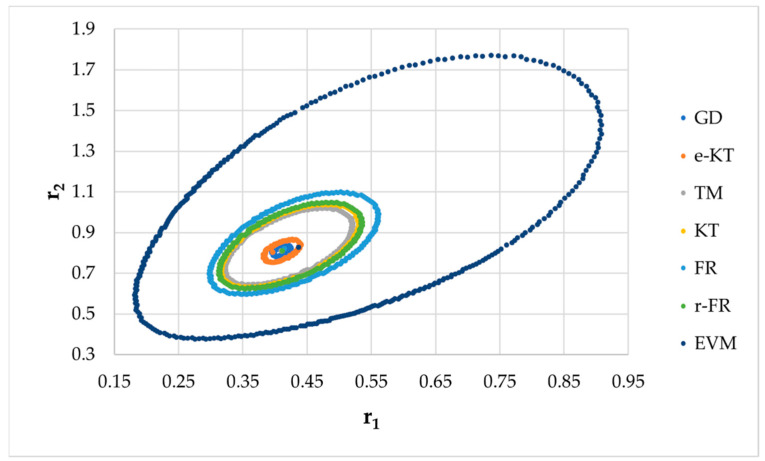
The JCR for following initial conditions *r*_1_ = 0.4, *r*_2_ = 0.8 *P_n_* ∊ (1–10%).

**Figure 19:**
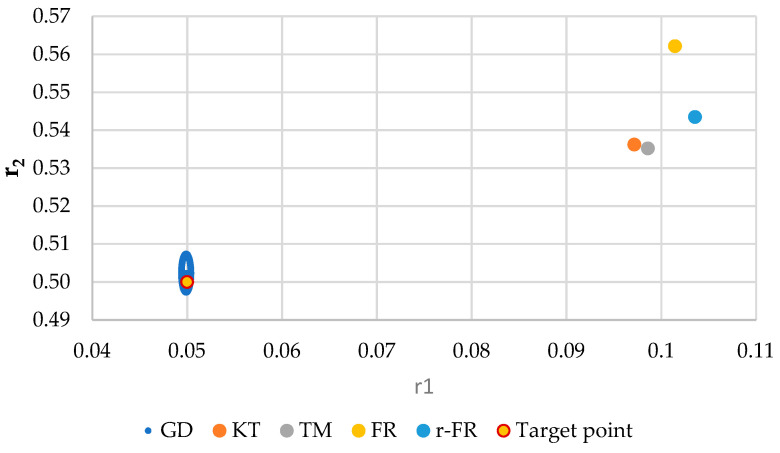
The JCR domain for initial data *r*_1_ = 0.05, *r*_2_ = 0.5 *P_n_* ∊ (30–55%).

**Figure 20:**
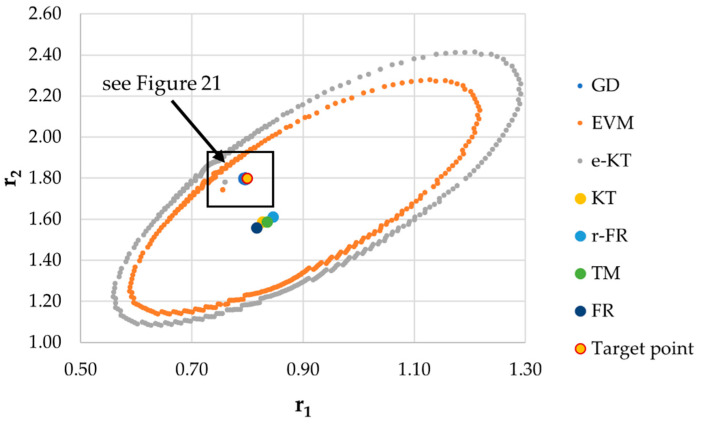
The JCR domain for initial data *r*_1_ = 0.8, *r*_2_ = 1.8 *P_n_* ∊ (30–55%).

**Figure 21:**
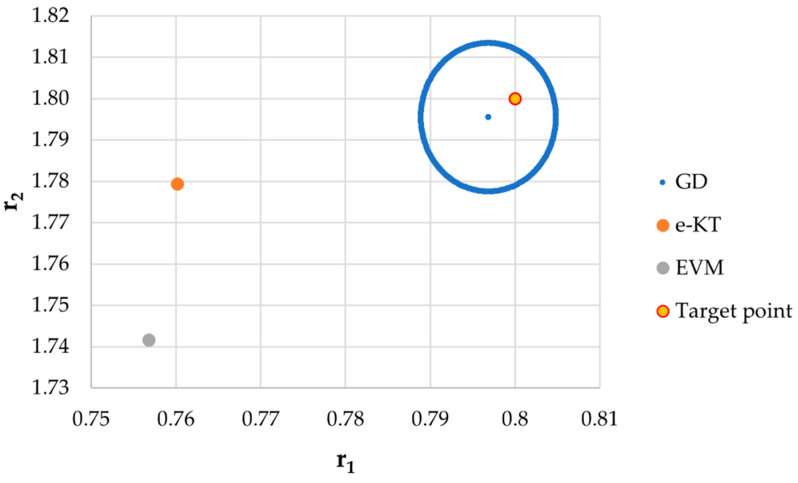
Details of JCR for GD given in Figure 20.

**Figure 22:**
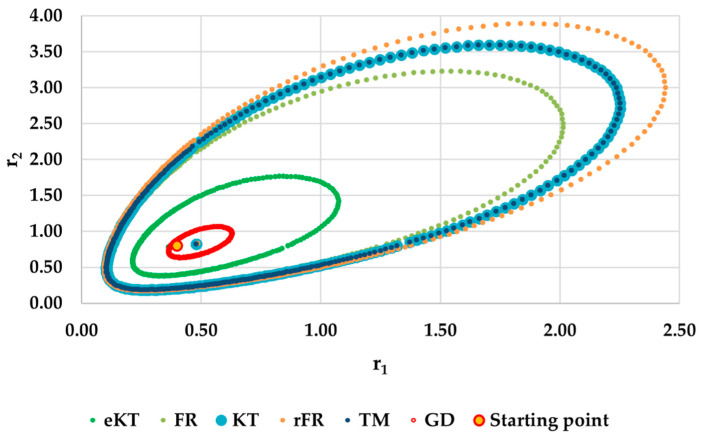
The JCR domain for initial data *r*_1_ = 0.40, *r*_2_ = 0.80 *P_n_* ∊ (30–55%).

**Figure 23:**
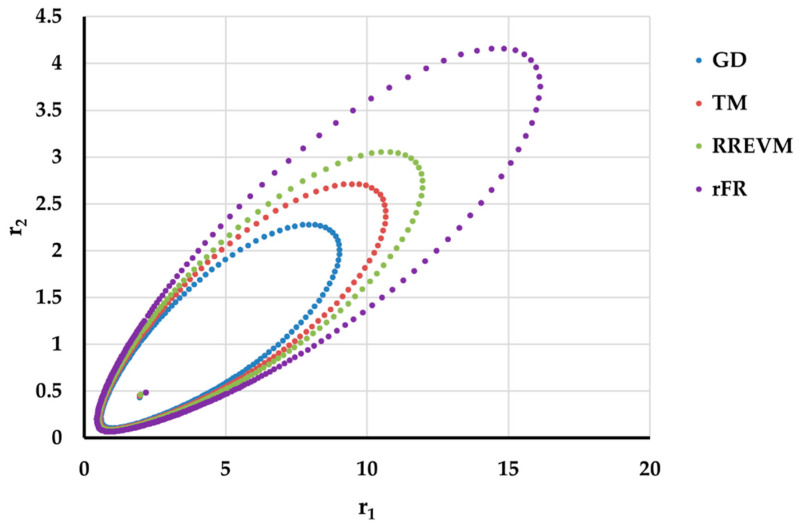
The JCR domain for copolymerization of n-butyl methacrylate with n-butyl acrylate [23].

**Figure 24:**
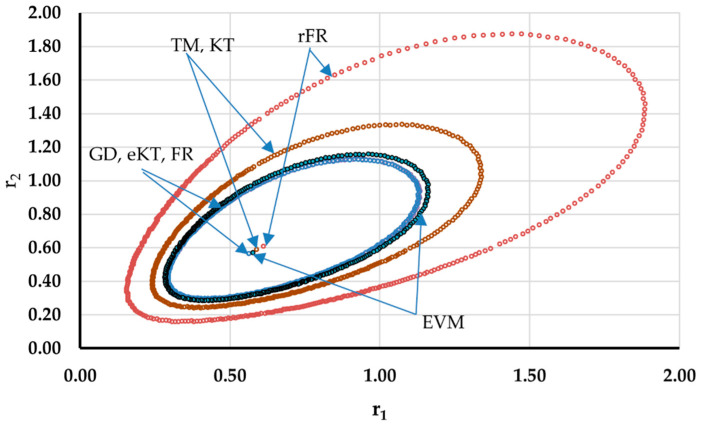
The JCR domain for copolymerization of 2-isopropenyl-2-oxazoline with methyl methacrylate [24].

**Figure 25:**
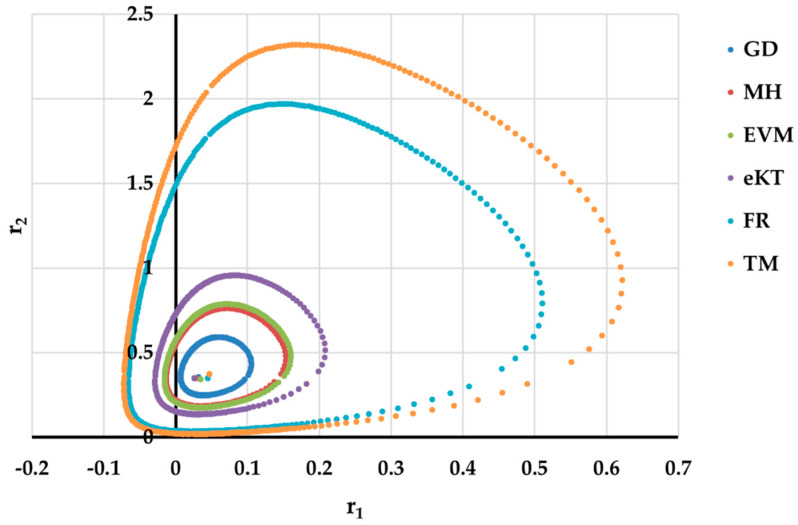
The JCR domain for copolymerization of N-(4-carboxyphenyl) maleimide with N-vinyl-2-pyrrolidone [25].

**Table 1:** Conditions imposed for the qualitative analysis of the methods taken in this analysis.

No.	*r*_1_	*r*_2_	*r*_1_ × *r*_2_	*P_n_*		
				*LC*	*MC*	*HC*
1	0.05	0.50	0.025	1–10	10–25	30–55
2	0.80	1.80	1.440	1–10	10–25	30–55
3	0.40	0.80	0.320	1–10	10–25	30–55

**Table 2:** Initial data for conversions between 1–10%.

*M*_1_	*P_n_*	*m*_1_		
		*LC1*	*LC2*	*LC3*
0.152	7.27	0.208	0.095	0.163
0.201	2.14	0.253	0.129	0.208
0.323	8.84	0.333	0.228	0.308
0.409	4.15	0.376	0.392	0.438
0.546	2.22	0.424	0.441	0.471
0.681	7.75	0.475	0.598	0.577
0.722	4.00	0.488	0.647	0.611
0.866	4.64	0.555	0.829	0.764
0.913	9.68	0.608	0.89	0.833

**Table 3:** Initial data for the conversions between 10–25%.

*M*_1_	*P_n_*	*m*_1_		
		*MC1*	*MC2*	*MC3*
0.170	15.23	0.222	0.110	0.180
0.209	20.56	0.254	0.141	0.215
0.310	17.83	0.325	0.222	0.299
0.453	18.48	0.396	0.354	0.407
0.587	14.75	0.445	0.493	0.505
0.663	20.91	0.473	0.583	0.568
0.707	12.65	0.486	0.632	0.601
0.857	11.34	0.555	0.819	0.756
0.935	18.73	0.670	0.919	0.873

**Table 4:** Initial data for conversions between 30–55%.

*M*_1_	*P_n_*	*m*_1_		
		1	2	3
0.199	32.18	0.243	0.137	0.205
0.272	47.25	0.297	0.203	0.269
0.313	44.14	0.325	0.237	0.303
0.415	31.50	0.381	0.324	0.381
0.577	49.66	0.451	0.501	0.517
0.672	50.77	0.488	0.607	0.588
0.768	38.42	0.523	0.716	0.671
0.851	44.52	0.589	0.818	0.766
0.915	34.67	0.666	0.895	0.849

**Table 5:** Reactivity ratios obtained for data *r*_1_ = 0.05, *r*_2_ = 0.50 *P_n_* ∊ (1–10%).

Method	*r*_1_	*r*_2_	*F^c^* × 1000	Bias	
				*r*_1_	*r*_2_
GD	0.0497	0.5032	1.1439	−0.0003	0.0032
e-KT	0.0467	0.5017	1.2610	−0.0033	0.0017
KT	0.0554	0.5080	3.2918	0.0054	0.0080
TM	0.0569	0.5090	4.0873	0.0069	0.0090
FR	0.0589	0.5313	5.5489	0.0089	0.0313
r-FR	0.0569	0.5076	6.2937	0.0069	0.0076
EVM *	0.0001	0.4980	35.9326	−0.0499	−0.0020

* the variant of EVM proposed by Chee and Ng [19].

**Table 6:** Reactivity ratios obtained for data *r*_1_ = 0.80, *r*_2_ = 1.80 *P_n_* ∊ (1–10%).

Method	*r*_1_	*r*_2_	*F^c^* × 1000	Bias	
				*r*_1_	*r*_2_
GD	0.7997	1.8052	0.2038	0.0003	−0.0052
e-KT	0.7963	1.8001	0.2957	0.0037	−0.0001
TM	0.8036	1.7787	1.4868	−0.0036	0.0213
FR	0.8054	1.7820	1.4906	−0.0054	0.0180
KT	0.8042	1.7785	1.5381	−0.0042	0.0215
r-FR	0.8034	1.7782	1.6146	−0.0034	0.0218
EVM *	1.244881	0.0001	219.1527	−0.4449	1.7999

* the variant of EVM proposed by Chee and Ng [19].

**Table 7:** Reactivity ratios obtained for data *r*_1_ = 0.40, *r*_2_ = 0.80 , *P_n_* ∊ (1–10%).

Method	*r*_1_	*r*_2_	*F^c^* × 1000	Bias	
				*r*_1_	*r*_2_
GD	0.3999	0.8016	0.2006	0.0001	−0.0016
e-KT	0.3974	0.8006	0.4229	0.0026	−0.0006
TM	0.4108	0.8076	1.5088	−0.0108	−0.0076
KT	0.4117	0.8081	1.6342	−0.0117	−0.0081
FR	0.4152	0.8199	1.9891	−0.0152	−0.0199
r-FR	0.4120	0.8079	2.3038	−0.0120	−0.0079
EVM *	0.4375	0.8268	4.9498	−0.0375	−0.0268

* the variant of EVM proposed by Chee and Ng [19].

**Table 8:** Reactivity ratios obtained for data *r*_1_ = 0.05, *r*_2_ = 0.5, *P_n_* ∊ (10–25%).

Method	*r*_1_	*r*_2_	*F^c^* × 1000	Bias	
				*r*_1_	*r*_2_
GD	0.0499	0.5017	0.1688	0.0001	−0.0017
e-KT	0.0439	0.4937	3.5836	0.0061	0.0063
KT	0.0679	0.5203	9.5071	−0.0179	−0.0203
TM	0.0710	0.5331	10.9795	−0.0210	−0.0331
FR	0.0762	0.5844	14.3585	−0.0262	−0.0844
r-FR	0.0718	0.5236	17.5811	−0.0218	−0.0236
EVM *	0.0555	0.0555	87.2141	−0.0055	0.4445

* the variant of EVM proposed by Chee and Ng [19].

**Table 9:** Reactivity ratios obtained for data *r*_1_ = 0.80, *r*_2_ = 1.80, *P_n_* ∊ (10–25%).

Method	*r*_1_	*r*_2_	*F^c^* × 1000	Bias	
				*r*_1_	*r*_2_
GD	0.7984	1.7992	0.1629	−0.0016	−0.0008
EVM *	0.7924	1.7897	0.3628	−0.0076	−0.0103
e-KT	0.7910	1.7867	0.4312	−0.0090	−0.0133
FR	0.8119	1.7157	4.4934	0.0118	−0.0843
KT	0.8085	1.7053	4.6748	0.0085	−0.0947
TM	0.8047	1.6973	4.7333	0.0047	−0.1027
r-FR	0.8145	1.7129	4.9533	0.0145	−0.0871

* the variant of EVM proposed by Chee and Ng [19].

**Table 10:** Reactivity ratios obtained for data *r*_1_ = 0.40, *r*_2_ = 0.80, *P_n_* ∊ (10–25%).

Method	*r*_1_	*r*_2_	*F^c^* × 1000	Bias	
				*r*_1_	*r*_2_
GD	0.3998	0.7991	0.2010	0.0002	0.0009
e-KT	0.3905	0.7927	1.0801	0.0095	0.0073
EVM *	0.4075	0.8261	1.7779	−0.0075	−0.0261
TM	0.4252	0.8072	3.5280	−0.0252	−0.0072
KT	0.4276	0.8098	3.7832	−0.0276	−0.0097
FR	0.4343	0.8033	4.2001	−0.0343	−0.0033
r-FR	0.4279	0.8095	4.9380	−0.0279	−0.0095

* the variant of EVM proposed by Chee and Ng [19].

**Table 11:** Reactivity ratios obtained for data *r*_1_ = 0.05, *r*_2_ = 0.50, *P_n_* ∊ (30–55%).

Methods	*r*_1_	*r*_2_	*F^c^* × 1000	Bias	
				*r*_1_	*r*_2_
GD	0.0499	0.5023	0.2557	0.0001	−0.0023
KT	0.0972	0.5362	21.3053	−0.0472	−0.0362
TM	0.0986	0.5352	21.8155	−0.0486	−0.0352
FR	0.1015	0.5621	22.7294	−0.0515	−0.0621
r-FR	0.1036	0.5435	34.2062	−0.0536	−0.0435
e-KT	0.0001	0.0001	108.9645	0.0499	0.4999
EVM *	0.0001	0.0001	108.9645	0.0499	0.4999

* the variant of EVM proposed by Chee and Ng [19].

**Table 12:** Reactivity ratios obtained for data *r*_1_ = 0.80, *r*_2_ = 1.80, *P_n_* ∊ (30–55%).

Methods	*r*_1_	*r*_2_	*F^c^* × 1000	Bias	
				*r*_1_	*r*_2_
GD	0.7969	1.7954	0.2326	0.0031	0.0046
EVM *	0.7568	1.7416	2.1460	0.0432	0.0584
e-KT	0.7602	1.7794	2.4694	0.0399	0.0207
KT	0.8277	1.5858	9.8426	−0.0277	0.2142
r-FR	0.8460	1.6112	10.2002	−0.0460	0.1888
TM	0.8354	1.5860	10.3112	−0.0354	0.2140
FR	0.8171	1.5577	10.3271	−0.0171	0.2423

* the variant of EVM proposed by Chee and Ng [19].

**Table 13:** Reactivity ratios obtained for data *r*_1_ = 0.40, *r*_2_ = 0.80, *P_n_* ∊ (30–55%).

Methods	*r*_1_	*r*_2_	*F^c^* × 1000	Bias	
				*r*_1_	*r*_2_
GD	0.4026	0.7920	1.8107	0.0026	−0.0080
e-KT	0.3648	0.7748	5.1504	−0.0352	−0.0252
FR	0.4699	0.7971	8.4630	0.0699	−0.0029
KT	0.4767	0.8170	8.5785	0.0767	0.0170
TM	0.4805	0.8187	8.9553	0.0805	0.0187
r-FR	0.4860	0.8289	12.0666	0.0860	0.0289
EVM *	0.3293	0.3292	42.4595	−0.0707	−0.4709

* the variant of EVM proposed by Chee and Ng [19].

**Table 14:** The reactivity ratios for copolymerization of n-butyl methacrylate with n-butyl acrylate [23].

Method	*r*_1_	*r*_2_	*F^c^* × 1000	Ref
GD	1.9839	0.4287	8.8713	This work
TM	1.9751	0.4480	9.4966	This work
r-FR	2.2045	0.4818	10.7046	This work
e-KT	2.3386	0.4965	12.3216	This work
KT	2.3222	0.5974	12.6241	This work
FR	2.8167	0.7535	27.4363	This work
EVM	2.6445	0.7768	30.6844	This work
RREVM	2.0080	0.4600	9.8015	[23]

**Table 15:** The reactivity ratios for copolymerization of N-vinyl prirrolidone with isobornyl methacrylate [24].

Method	*r*_1_	*r*_2_	*F^c^* × 1000	Ref
GD	0.5644	0.5626	3.4453	This work
eKT	0.5630	0.5649	3.4536	This work
FR	0.5696	0.5624	3.4996	This work
EVM	0.5765	0.5694	3.5844	This work
KT	0.5879	0.5895	4.2869	This work
TM	0.5899	0.5830	4.2917	This work
rFR	0.6122	0.6061	5.8456	This work
OPT	0.71	0.63	13.5698	[24]

**Table 16:** The reactivity ratios for copolymerization of NCPM with NVP [25].

Method	*r*_1_	*r*_2_	*F^c^* × 1000	Ref.
GD	0.0327	0.3521	2.3722	this work
EVM	0.0352	0.3404	3.9834	this work
e-KT	0.0269	0.3467	5.0836	this work
FR	0.0452	0.3472	9.1372	this work
TM	0.0474	0.3722	10.0274	this work
KT	0.0592	0.3934	16.9579	this work
e-KT	0.0270	0.3470	5.0942	[24]
MH	0.0290	0.3470	3.7893	[24]

## Data Availability

Data sharing not applicable.
